# Supplementary material for: Splitting on categorical predictors in random forests
Source: PeerJ. 2019 Feb 7;7:e6339. doi: 10.7717/peerj.6339 (PMC6368971; doi:10.7717/peerj.6339)
Supplement: Supplemental Information 1 [file peerj-07-6339-s001.pdf]

# Supplementary material for "Splitting on categorical predictors in random forests"

Marvin N. Wright and Inke R. König

| Predictor type | Outcome type | Nominal predictor method | | | |
|---|---|---|---|---|---|
| | | Order (split) | Order (once) | Dummy | Ignore |
| Digit | Regression | $9.6 \times 10^{-01}$ | $\mathbf{3.9 \times 10^{-21}}$ | $\mathbf{9.7 \times 10^{-55}}$ | $\mathbf{8.4 \times 10^{-71}}$ |
| | Binary Class. | $3.5 \times 10^{-01}$ | $\mathbf{7.4 \times 10^{-08}}$ | $\mathbf{6.1 \times 10^{-17}}$ | $\mathbf{7.4 \times 10^{-39}}$ |
| | Multiclass Class. | $1.7 \times 10^{-01}$ | $\mathbf{5.1 \times 10^{-37}}$ | $\mathbf{6.8 \times 10^{-77}}$ | $\mathbf{4.1 \times 10^{-80}}$ |
| | Survival | $\mathbf{5.6 \times 10^{-16}}$ | $\mathbf{4.2 \times 10^{-02}}$ | $\mathbf{3.1 \times 10^{-23}}$ | $\mathbf{2.9 \times 10^{-08}}$ |
| SNP | Regression | $6.8 \times 10^{-01}$ | $\mathbf{2.2 \times 10^{-17}}$ | $\mathbf{6.3 \times 10^{-24}}$ | $\mathbf{4.1 \times 10^{-40}}$ |
| | Binary Class. | $1.2 \times 10^{-01}$ | $9.7 \times 10^{-01}$ | $3.8 \times 10^{-01}$ | $\mathbf{1.3 \times 10^{-06}}$ |
| | Multiclass Class. | $9.9 \times 10^{-01}$ | $\mathbf{9.8 \times 10^{-15}}$ | $\mathbf{4.9 \times 10^{-19}}$ | $\mathbf{3.6 \times 10^{-40}}$ |
| | Survival | $4.0 \times 10^{-01}$ | $3.5 \times 10^{-01}$ | $\mathbf{7.2 \times 10^{-03}}$ | $\mathbf{1.4 \times 10^{-02}}$ |
| Group | Regression | $5.1 \times 10^{-01}$ | $4.1 \times 10^{-01}$ | $\mathbf{1.1 \times 10^{-07}}$ | $\mathbf{4.0 \times 10^{-23}}$ |
| | Binary Class. | $6.6 \times 10^{-01}$ | $7.7 \times 10^{-01}$ | $6.5 \times 10^{-01}$ | $\mathbf{4.9 \times 10^{-06}}$ |
| | Multiclass Class. | $1.6 \times 10^{-01}$ | $6.6 \times 10^{-02}$ | $\mathbf{4.3 \times 10^{-33}}$ | $\mathbf{1.3 \times 10^{-29}}$ |
| | Survival | $1.8 \times 10^{-01}$ | $\mathbf{9.8 \times 10^{-04}}$ | $\mathbf{1.1 \times 10^{-28}}$ | $\mathbf{2.4 \times 10^{-02}}$ |

Table S1: $p$-values of simulations for $n = 100$. Each $p$-value corresponds to a two-sided paired $t$-test, compared to the Partition method. Significant results at $\alpha = 0.05$ are highlighted in bold.

| Predictor type | Outcome type | Nominal predictor method | | | |
|---|---|---|---|---|---|
| | | Order (split) | Order (once) | Dummy | Ignore |
| Digit | Regression | $2.1 \times 10^{-01}$ | $\mathbf{1.8 \times 10^{-09}}$ | $\mathbf{6.0 \times 10^{-20}}$ | $\mathbf{1.5 \times 10^{-48}}$ |
| | Binary Class. | $9.3 \times 10^{-01}$ | $3.8 \times 10^{-01}$ | $\mathbf{3.0 \times 10^{-09}}$ | $\mathbf{6.7 \times 10^{-21}}$ |
| | Multiclass Class. | $4.1 \times 10^{-01}$ | $\mathbf{1.3 \times 10^{-43}}$ | $\mathbf{1.9 \times 10^{-34}}$ | $\mathbf{2.5 \times 10^{-55}}$ |
| | Survival | $\mathbf{2.0 \times 10^{-07}}$ | $1.5 \times 10^{-01}$ | $\mathbf{9.1 \times 10^{-13}}$ | $\mathbf{5.7 \times 10^{-04}}$ |
| SNP | Regression | $9.4 \times 10^{-01}$ | $\mathbf{1.6 \times 10^{-09}}$ | $\mathbf{2.3 \times 10^{-13}}$ | $\mathbf{3.0 \times 10^{-21}}$ |
| | Binary Class. | $8.0 \times 10^{-01}$ | $7.0 \times 10^{-01}$ | $7.1 \times 10^{-01}$ | $1.5 \times 10^{-01}$ |
| | Multiclass Class. | $8.5 \times 10^{-01}$ | $\mathbf{1.1 \times 10^{-17}}$ | $\mathbf{2.6 \times 10^{-23}}$ | $\mathbf{6.8 \times 10^{-16}}$ |
| | Survival | $1.7 \times 10^{-01}$ | $3.8 \times 10^{-01}$ | $3.8 \times 10^{-01}$ | $\mathbf{3.2 \times 10^{-02}}$ |
| Group | Regression | $2.4 \times 10^{-01}$ | $3.6 \times 10^{-01}$ | $\mathbf{9.6 \times 10^{-05}}$ | $\mathbf{3.4 \times 10^{-10}}$ |
| | Binary Class. | $1.3 \times 10^{-01}$ | $8.3 \times 10^{-01}$ | $2.7 \times 10^{-01}$ | $\mathbf{1.8 \times 10^{-04}}$ |
| | Multiclass Class. | $6.8 \times 10^{-01}$ | $\mathbf{1.0 \times 10^{-02}}$ | $\mathbf{5.4 \times 10^{-10}}$ | $\mathbf{4.8 \times 10^{-10}}$ |
| | Survival | $\mathbf{4.7 \times 10^{-02}}$ | $4.6 \times 10^{-01}$ | $\mathbf{7.3 \times 10^{-16}}$ | $\mathbf{2.9 \times 10^{-02}}$ |

Table S2: $p$-values of simulations for $n = 50$. Each $p$-value corresponds to a two-sided paired $t$-test, compared to the Partition method. Significant results at $\alpha = 0.05$ are highlighted in bold.

| Predictor type | Outcome type | Nominal predictor method | | | |
|---|---|---|---|---|---|
| | | Order (split) | Order (once) | Dummy | Ignore |
| Digit | Regression | $9.5 \times 10^{-01}$ | $\mathbf{2.0 \times 10^{-42}}$ | $\mathbf{1.8 \times 10^{-74}}$ | $\mathbf{5.8 \times 10^{-91}}$ |
| | Binary Class. | $3.6 \times 10^{-01}$ | $\mathbf{3.2 \times 10^{-20}}$ | $\mathbf{3.4 \times 10^{-40}}$ | $\mathbf{5.2 \times 10^{-62}}$ |
| | Multiclass Class. | $4.5 \times 10^{-01}$ | $\mathbf{1.8 \times 10^{-19}}$ | $\mathbf{2.6 \times 10^{-86}}$ | $\mathbf{5.0 \times 10^{-108}}$ |
| | Survival | $\mathbf{4.2 \times 10^{-14}}$ | $7.6 \times 10^{-01}$ | $\mathbf{1.3 \times 10^{-45}}$ | $\mathbf{1.5 \times 10^{-06}}$ |
| SNP | Regression | $4.5 \times 10^{-01}$ | $\mathbf{7.2 \times 10^{-16}}$ | $\mathbf{2.4 \times 10^{-21}}$ | $\mathbf{1.0 \times 10^{-37}}$ |
| | Binary Class. | $2.9 \times 10^{-01}$ | $8.1 \times 10^{-01}$ | $5.4 \times 10^{-01}$ | $\mathbf{5.3 \times 10^{-04}}$ |
| | Multiclass Class. | $1.8 \times 10^{-01}$ | $\mathbf{4.9 \times 10^{-05}}$ | $\mathbf{9.0 \times 10^{-08}}$ | $\mathbf{2.2 \times 10^{-50}}$ |
| | Survival | $4.8 \times 10^{-01}$ | $9.9 \times 10^{-01}$ | $\mathbf{2.6 \times 10^{-07}}$ | $\mathbf{6.2 \times 10^{-04}}$ |
| Group | Regression | $5.5 \times 10^{-01}$ | $\mathbf{2.1 \times 10^{-02}}$ | $\mathbf{4.5 \times 10^{-11}}$ | $\mathbf{1.8 \times 10^{-32}}$ |
| | Binary Class. | $2.3 \times 10^{-01}$ | $\mathbf{3.1 \times 10^{-04}}$ | $\mathbf{3.7 \times 10^{-05}}$ | $\mathbf{1.1 \times 10^{-17}}$ |
| | Multiclass Class. | $3.3 \times 10^{-01}$ | $\mathbf{1.4 \times 10^{-17}}$ | $\mathbf{1.4 \times 10^{-62}}$ | $\mathbf{1.3 \times 10^{-50}}$ |
| | Survival | $\mathbf{6.5 \times 10^{-05}}$ | $\mathbf{7.4 \times 10^{-04}}$ | $\mathbf{4.9 \times 10^{-29}}$ | $\mathbf{3.0 \times 10^{-06}}$ |

Table S3: $p$-values of simulations for $n = 200$. Each $p$-value corresponds to a two-sided paired $t$-test, compared to the Partition method. Significant results at $\alpha = 0.05$ are highlighted in bold.

| Dataset | Nominal predictor method | | | |
|---|---|---|---|---|
| | Order (split) | Order (once) | Dummy | Ignore |
| Tic-tac-toe | $9.5 \times 10^{-01}$ | $8.1 \times 10^{-01}$ | $9.4 \times 10^{-01}$ | $5.1 \times 10^{-02}$ |
| Splice | $8.5 \times 10^{-01}$ | $7.8 \times 10^{-01}$ | $1.8 \times 10^{-01}$ | $\mathbf{1.5 \times 10^{-02}}$ |
| MPG | NA* | $7.8 \times 10^{-01}$ | $9.3 \times 10^{-02}$ | $\mathbf{4.4 \times 10^{-02}}$ |
| Servo | $9.5 \times 10^{-01}$ | $8.1 \times 10^{-01}$ | $\mathbf{3.2 \times 10^{-02}}$ | $\mathbf{2.5 \times 10^{-02}}$ |
| RA SNPs | $9.0 \times 10^{-01}$ | $8.6 \times 10^{-01}$ | $8.3 \times 10^{-01}$ | $2.9 \times 10^{-01}$ |
| AIDS | $8.8 \times 10^{-01}$ | $8.2 \times 10^{-01}$ | $5.6 \times 10^{-01}$ | $7.3 \times 10^{-01}$ |

Table S4: $p$-values of real data analyses. Each $p$-value corresponds to a two-sided corrected paired $t$-test (Nadeau and Bengio, 2003), compared to the Partition method. Significant results at $\alpha = 0.05$ are highlighted in bold. *No results could be obtained for the Partition method on the MPG dataset because the category limit was reached. The other methods are compared to the Order (split) method instead.

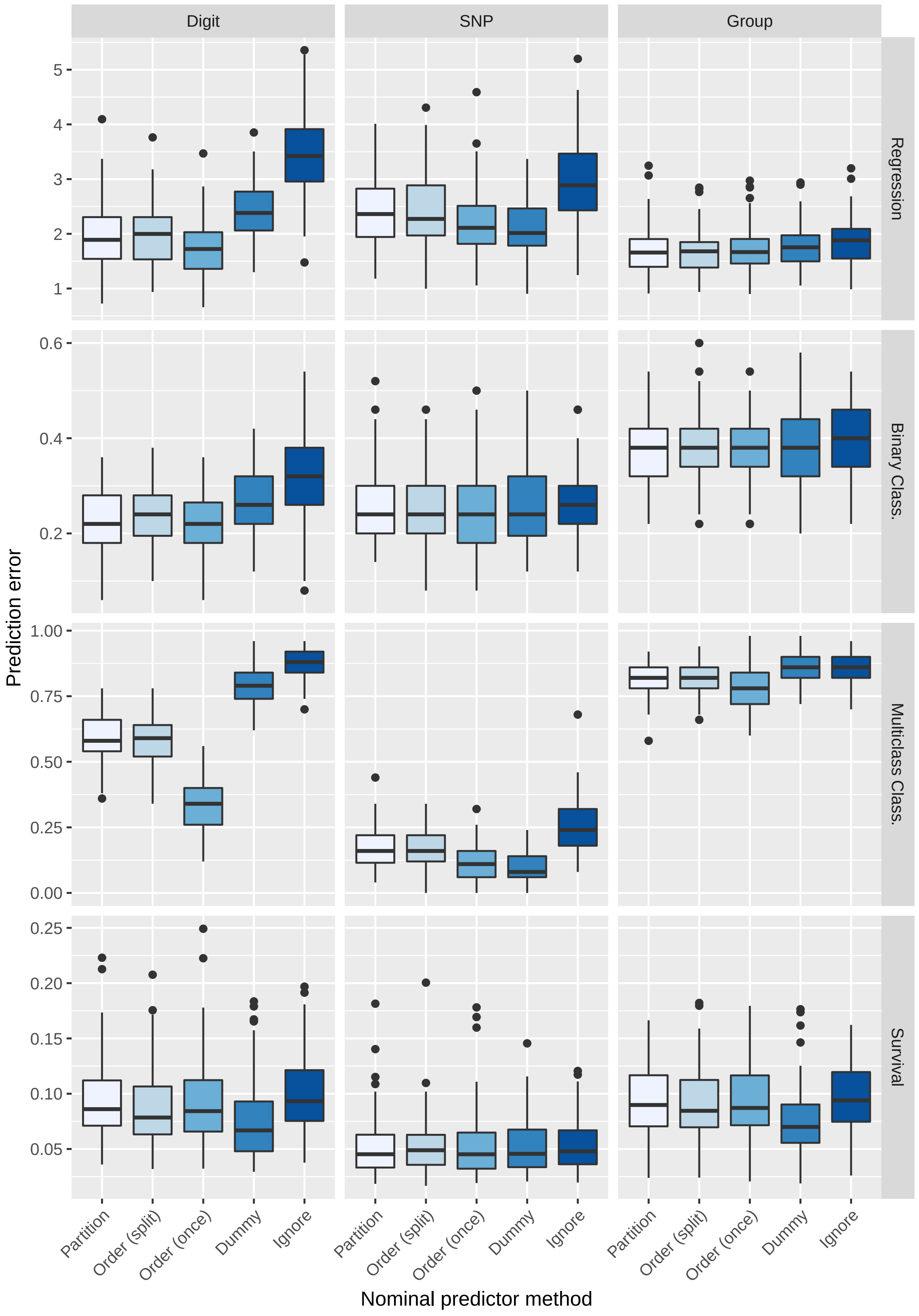


Figure S1: Simulation results for $n = 50$. The vertical panels correspond to the predictor type (Digit, SNP or Group), the horizontal panels to the outcome type (regression, binary classification, multiclass classification or survival). Each boxplot represents the prediction error for one method to handle nominal predictor variables, applied to one simulation scenario. The prediction error is measured by the mean squared error for regression, by the proportion of misclassifications for classification and by the integrated Brier score for survival.

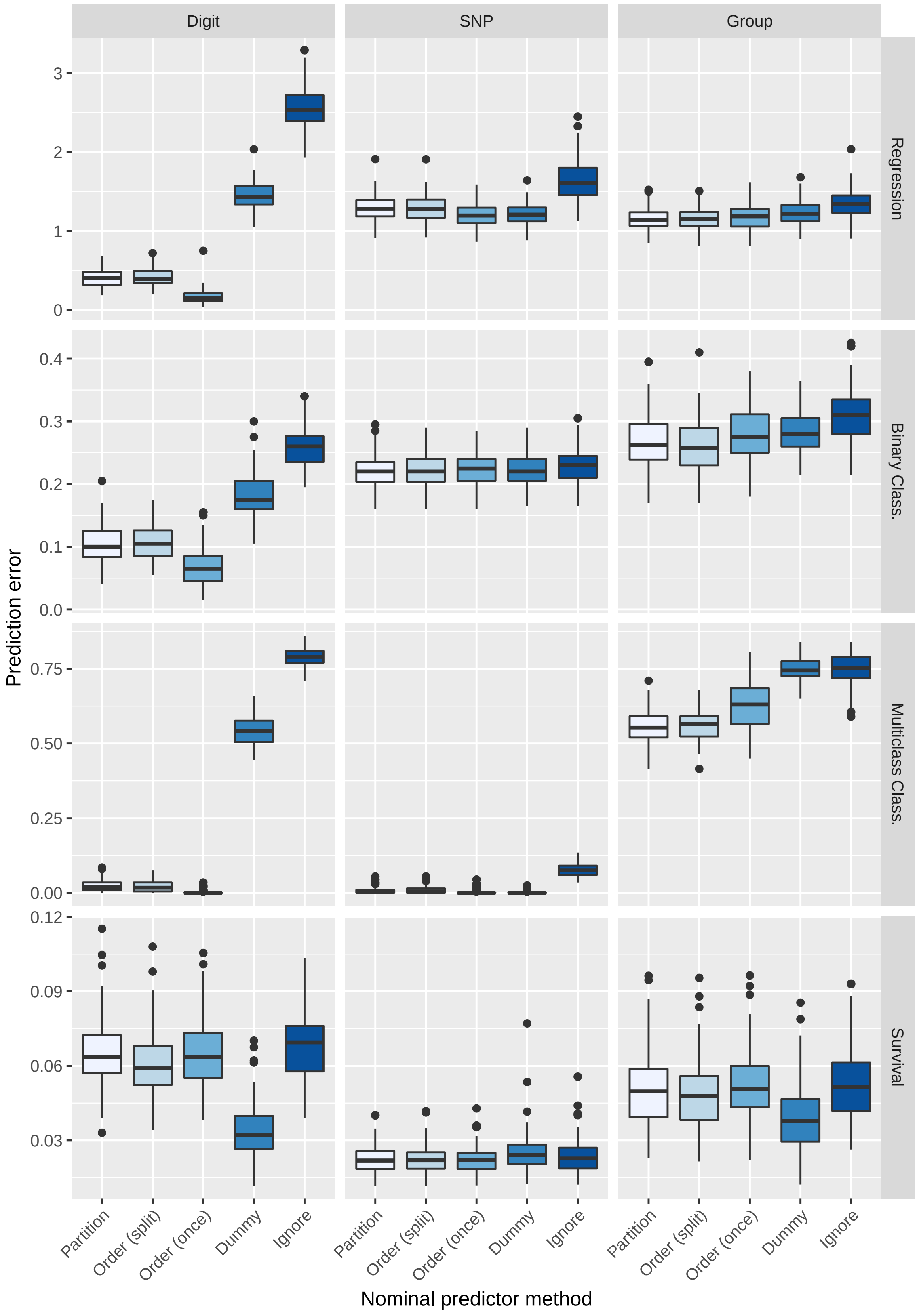


Figure S2: Simulation results for $n = 200$. The vertical panels correspond to the predictor type (Digit, SNP or Group), the horizontal panels to the outcome type (regression, binary classification, multiclass classification or survival). Each boxplot represents the prediction error for one method to handle nominal predictor variables, applied to one simulation scenario. The prediction error is measured by the mean squared error for regression, by the proportion of misclassifications for classification and by the integrated Brier score for survival.

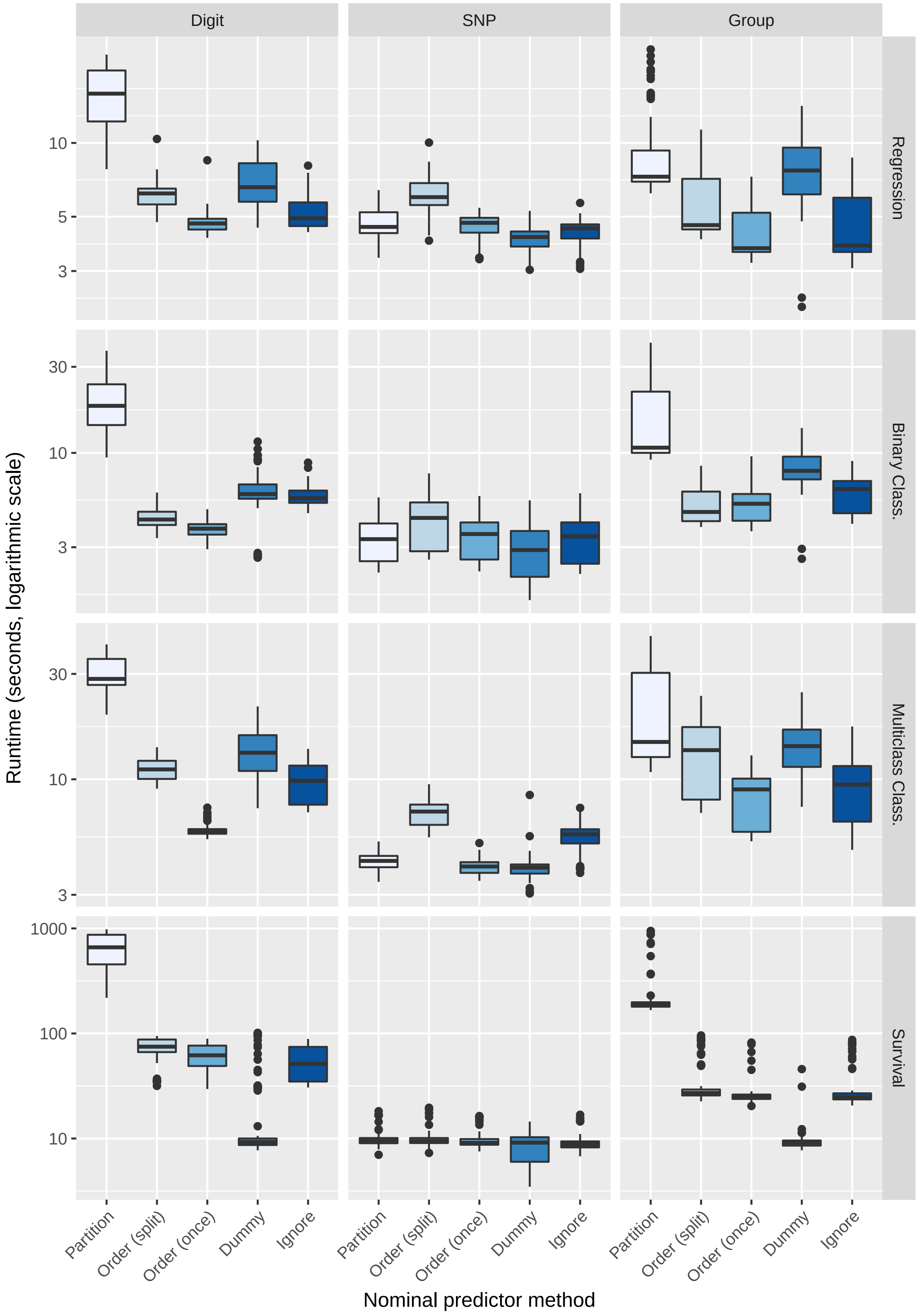


Figure S3: Runtimes of simulations. The vertical panels correspond to the predictor type (Digit, SNP or Group), the horizontal panels to the outcome type (regression, binary classification, multiclass classification or survival). Each boxplot represents the runtime (in seconds) for one method to handle nominal predictor variables, applied to one simulation scenario. The vertical axis is displayed on a logarithmic scale.

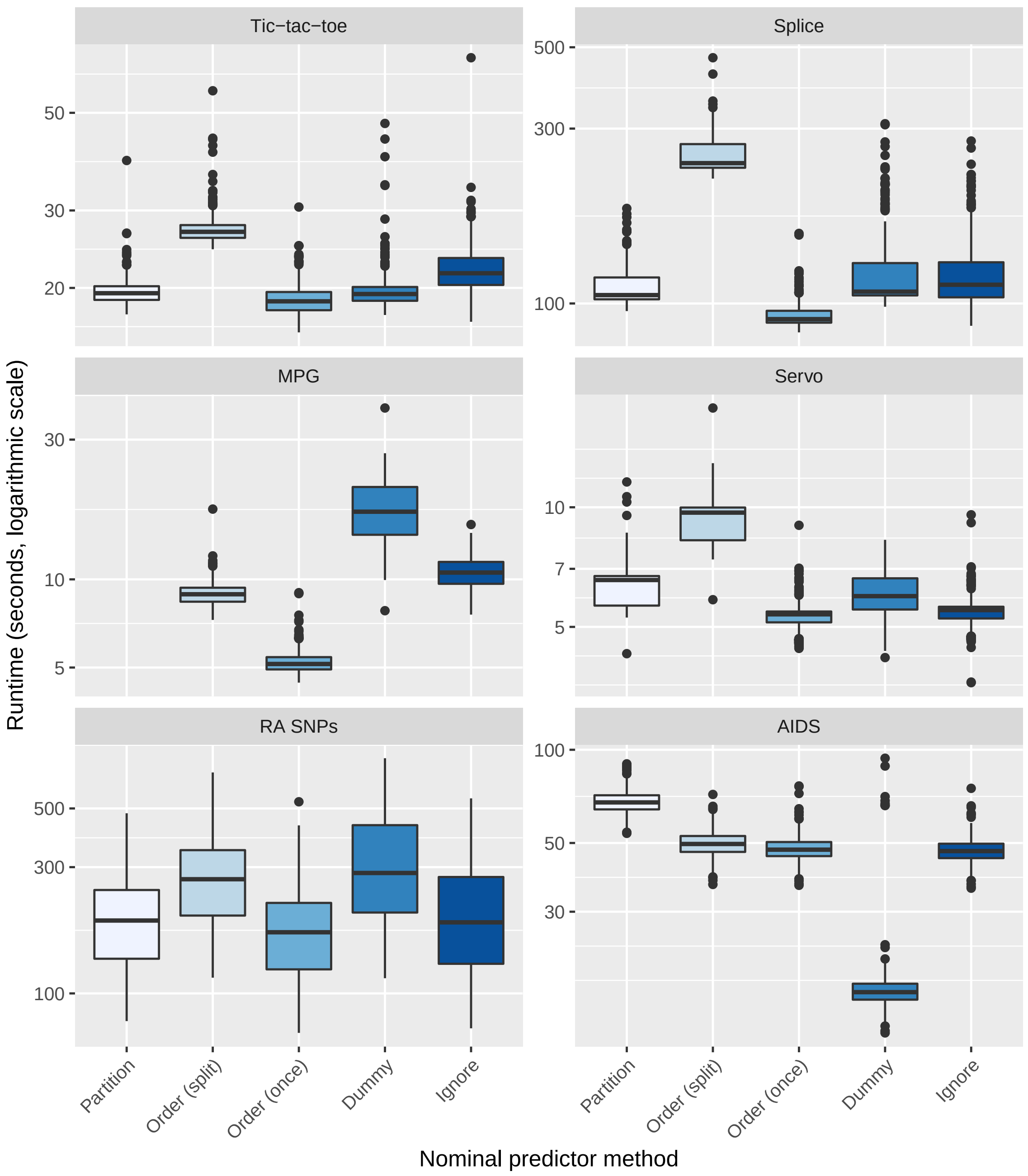


Figure S4: Runtimes of real data analyses. The panels correspond to the datasets. Each boxplot represents the runtime (in seconds) of one method to handle nominal predictor variables, applied to one dataset. No results could be obtained for the Partition method on the MPG dataset because the category limit was reached. The vertical axis is displayed on a logarithmic scale.
